# A case of pancreatic neuroendocrine tumor in a patient with neurofibromatosis-1

**DOI:** 10.1186/1477-7819-10-153

**Published:** 2012-07-23

**Authors:** Takeshi Nishi, Yasunari Kawabata, Youko Hari, Hiroshi Imaoka, Noriyoshi Ishikawa, Seiji Yano, Riruke Maruyama, Yoshitsugu Tajima

**Affiliations:** 1 Department of Digestive and General Surgery, Shimane University Faculty of Medicine, 89-1 Enyacho, Izumo, 693-8501, Shimane, Japan; 2 Department of Gastroenterology and Hepatology, Shimane University Faculty of Medicine, Shimane, Japan; 3 Department of Organ Pathology, Shimane University Faculty of Medicine, Shimane, Japan

**Keywords:** Neuroendocrine tumor, Neurofibromatosis-1, Neurofibromin, Pancreatic acinar-endocrine carcinoma, Pancreatic neuroendocrine tumor, von Recklinghausen’s disease

## Abstract

Patients with neurofibromatosis-1 (NF-1) sometime develop neuroendocrine tumors (NET). Although these NETs usually occur in the duodenum or peri-ampullary region, they occasionally grow in the pancreas (PNET). A 62-year-old man with NF-1 had mild liver dysfunction and was admitted to our hospital for further examination. An abdominal contrast-enhanced computed tomography scan demonstrated a 30-mm tumor in the head of the pancreas. The scan showed an invasion of the tumor into the duodenum, and biopsy under an endoscopic ultrasonography indicated that the tumor was a NET. A subtotal stomach-preserving pancreaticoduodenectomy was performed. Macroscopically, the pancreatic tumor was white and elastic hard. Microscopically, tumor cells were composed of ribbons, cords, and solid nests with an acinus-like structure. The tumor was diagnosed as NET G2 according to the WHO classification (2010). The product of the*NF-1* gene, i.e., neurofibromin, was weakly positive in the tumor cells, suggesting that the tumor was induced by a mutation in the *NF-1* gene. This is the seventh case of PNET arising in NF-1 patients worldwide.

## Background

Neurofibromatosis-1 (NF-1), known as von Recklinghausen’s disease, is an autosomal dominant, multisystem disorder that occurs in 1 in 2,500–3,000 live births [1,2]. This hereditary disorder is characterized by pigmentary features (café-au-lait macules, skinfold freckling), neurofibroma, orthopedic features (scoliosis, dysplasia of a long bone), and ophthalmologic features (Lisch nodules, optic glioma) [1-3].

The *NF-1* gene is located on chromosome 17q11.2, and encodes the protein neurofibromin [4,5]. Neurofibromin is a tumor suppressor expressed in many cells, so *NF-1* gene mutation leads to uncontrolled cell proliferation and development of benign and malignant tumors, including neuroendocrine tumors (NETs) [1,3]. The most frequent target organ of NETs in patients with NF-1 is the duodenum and peri-ampullary region [6-9]. Pancreatic NETs (PNETs) sometimes occur in hereditary diseases, such as multiple endocrine neoplasia type 1 (MEN-1) and von Hippel-Lindau disease (VHL), but they rarely develop in patients with NF-1 [10,11]. Only six cases of PNETs, including four malignant cases of PNETs, have previously been reported in patients with NF-1 [12-17]. Here, we report a rare case of PNET arising in a patient with NF-1.

## Case presentation

**Figure 1:**
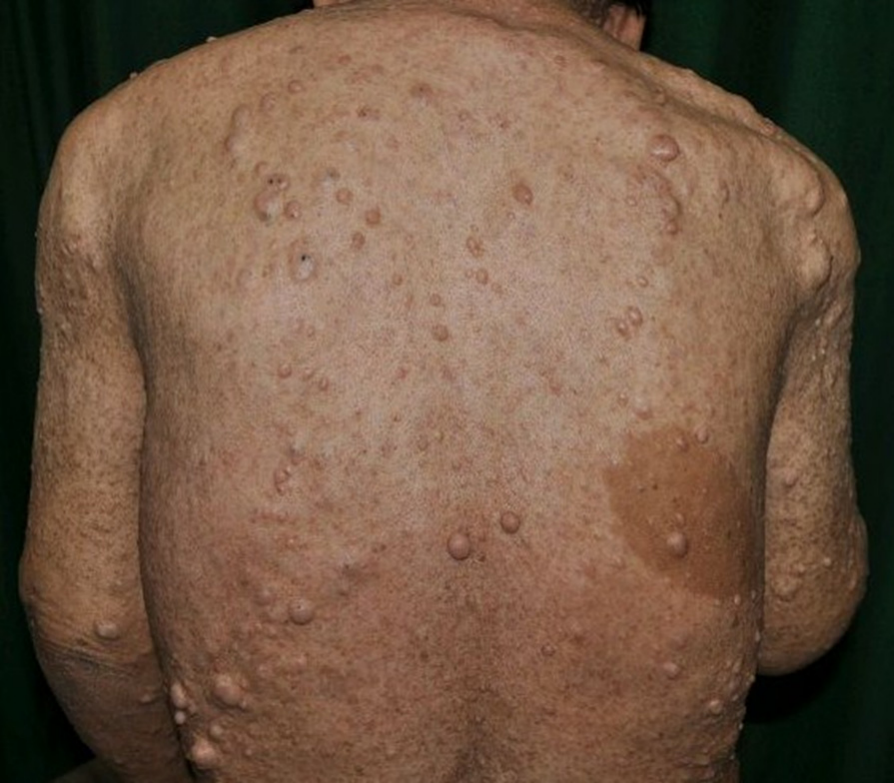
**A view of the back of the patient.** Numerous café-au-lait macules and cutaneous neurofibromas can be seen.

**Figure 2:**
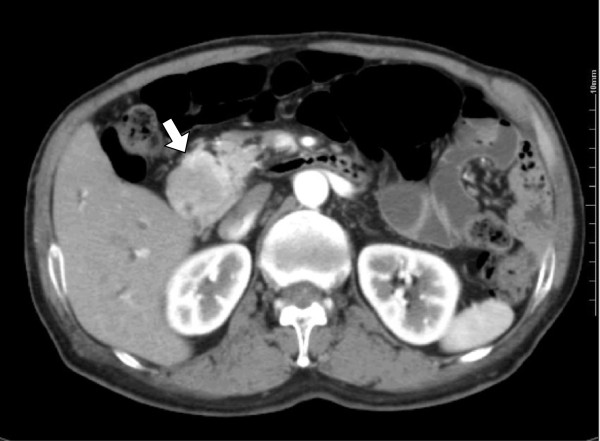
**Findings of an abdominal contrast-enhanced computed tomography scan.** The scan demonstrates a 30-mm tumor in the head of the pancreas (*arrow*), with slow enhancement.

A 62-year-old man with NF-1 had mild liver dysfunction diagnosed by medical examination, and he was admitted to our hospital for further examination. In his past history, he had developed blindness in childhood because of pigmentary retinal dystrophy. There was no history of hypoglycemia or peptic ulcer. On admission, he had no clinical symptoms. His height was 141 cm and his body weight was 52 kg. Numerous café-au-lait macules and cutaneous neurofibromas were noted over his whole body (Figure 1). There were no palpable lymph nodes in the surface or mass lesions in the abdomen. The patient’s blood pressure was 112/70 mmHg and heart rate was 72 beats per min without abnormality on electrocardiogram examination. Alkaline phosphatase, leucine aminopeptidase, and γ-glutamyl transpeptidase levels were slightly elevated; other biochemical data, including tumor markers, were almost within the normal ranges. Values for fasting plasma glucose, hemoglobin A1c, and serum glucagon were within the normal limits, but the serum gastrin level was slightly higher than the normal upper limit. Upper gastrointestinal endoscopy showed a reddish, chorioepithelial hyperplasia of the second part of the duodenum. A biopsy specimen of the lesion revealed hyperplasia of Brunner’s glands. There was no peptic ulcer in the stomach and duodenum. Colonoscopy showed no abnormality. An abdominal contrast-enhanced computed tomography (CT) scan demonstrated a low-attenuating tumor in the head of the pancreas, measuring 30 mm in diameter, with slow enhancement (Figure 2). The upper common bile duct was extended, but the main pancreatic duct was not dilated. Some lymph nodes around the hepatic hilum were swollen, with a maximal diameter of 25 mm. No tumor was detected in the liver and lung, or in the pituitary and parathyroid glands. Endoscopic retrograde cholangiopancreatography (ERCP) showed that the lower common bile duct and main pancreatic duct were oppressed without any encroachment. Endoscopic ultrasonography (EUS) showed a low echoic tumor, measuring 35 mm in diameter, near the papilla of Vater, compressing both the common bile duct and main pancreatic duct. The lesion continued to the fourth layer of the duodenal wall. Swollen lymph nodes were detected near the common bile duct. Fine-needle aspiration biopsy under EUS revealed that the pancreatic mass was a neuroendocrine tumor, compatible with a diagnosis of PNET. A subtotal stomach-preserving pancreaticoduodenectomy was performed. At laparotomy, there was no fluid collection, peritoneal dissemination, or liver metastasis. Because we found two small nodules in the jejunum and one small nodule in the stomach, partial resection of the jejunal wall and gastric wall was performed.

**Figure 3:**
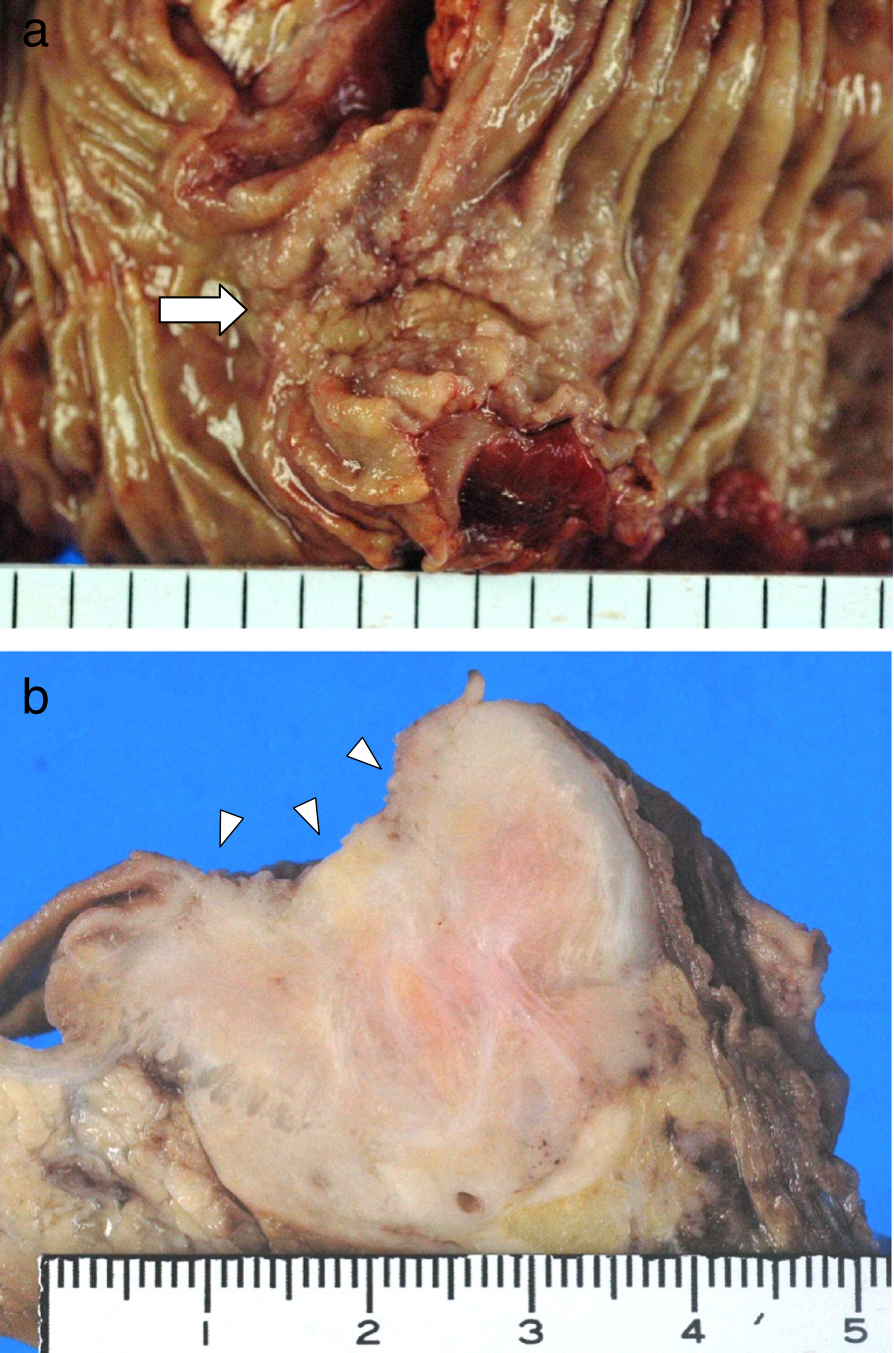
**Macroscopic findings of the pancreatic tumor.** (**a**) The tumor showed an invasion into the duodenal mucosa (arrow). (**b**) A white and elastic hard tumor, 30 × 35 × 40 mm in size, was located in the head of the pancreas with direct invasion into the duodenum (arrowheads).

**Figure 4:**
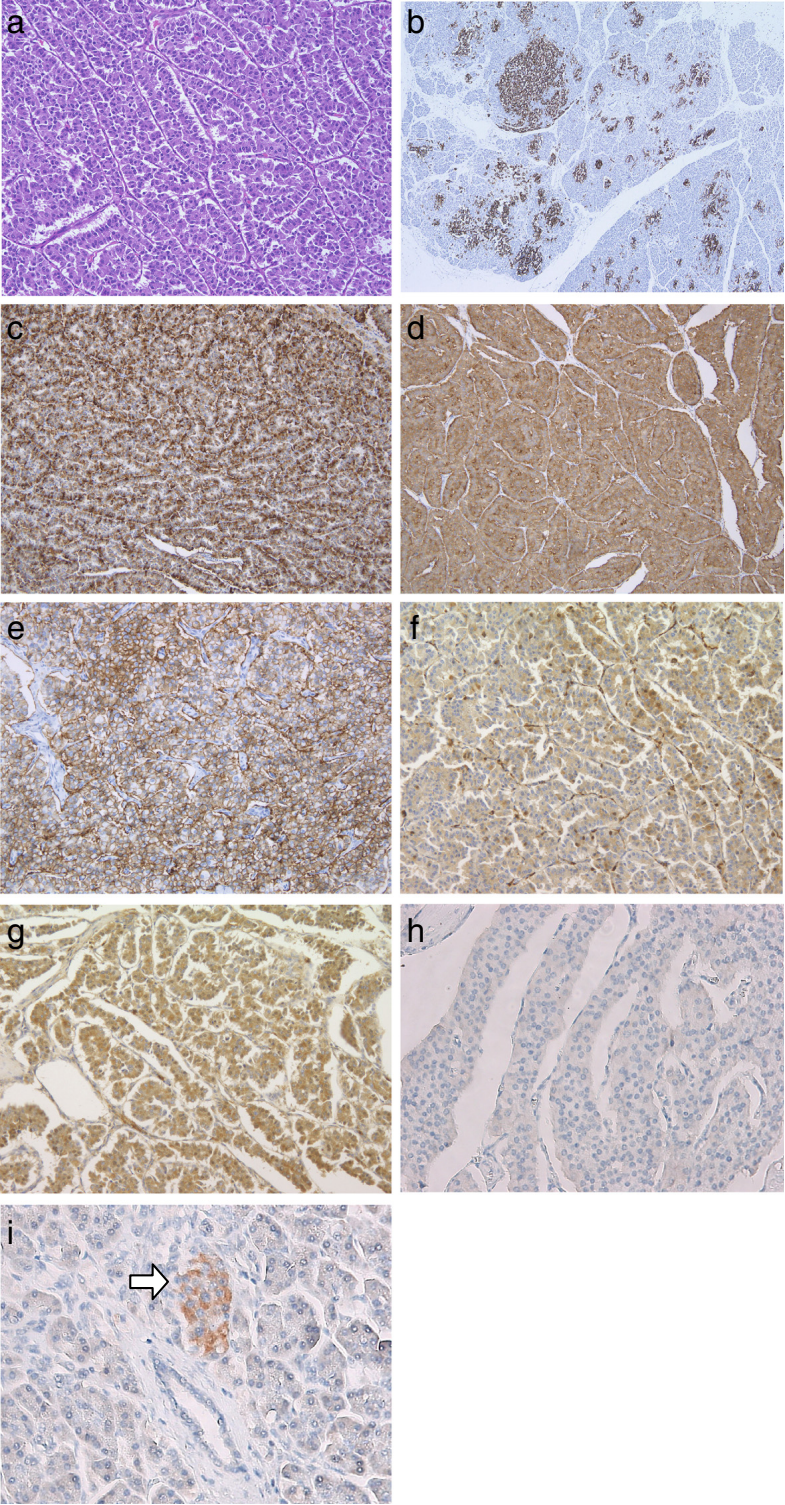
**Microscopic findings of the pancreatic tumor.** (**a**) Tumor cells were composed of ribbons, cords, and solid nests with acinus-like structure (hematoxylin and eosin, magnification 200×). (**b**) Hyperplasia of islet cells (positive for chromogranin A staining) was frequently found in the non-neoplastic pancreas (chromogranin A staining, magnification 140×). (**c**,**d**,**e**) Expression of chromogranin A, synaptophysin, and CD56 was strongly positive in the tumor cells (**c**: chromogranin A staining, magnification 400×; **d**: synaptophysin staining, magnification 400×; **e**: CD56 staining, magnification 400×). (**f**/**g**) Some of tumor cells, particularly in the acinus-like structure, were positive for α1-antichymotrypsin and α1-antitrypsin (f: α1-antichymotrypsin staining, magnification 400×/g: α1-antitrypsin staining, magnification 400×). (**h**) Expression of neurofibromin was negative in the tumor cells (neurofibromin staining, magnification 400×). (**i**) Pancreatic islet cells in the non-neoplastic region demonstrated a strongly positive expression of neurofibromin (arrow). Acinar cells and ductal epithelial cells showed negative expression of neurofibromin (neurofibromin staining, magnification 400×).

Macroscopically, a white and elastic hard tumor, 30 × 35 × 40 mm in size, was identified in the head of the pancreas, and it had invaded the duodenum (Figure 3). Microscopically, tumor cells were composed of ribbons, cords, and solid nests with an acinus-like structure (Figure 4a). Tumor cells were considered to have islet cell origin because they had uniform nuclei with a salt-and-pepper appearance, and hyperplasia of islet cells was frequently found in the non-neoplastic pancreas (Figure 4b). On the other side, in the part of acinus-like pattern mimicking normal acinar cells, cytoplasms of these cells were eosinophilic and granular, associated with zymogen granules, which were positive for α-antitrypsin and α-antichymotrypsin. These tumor cells were smaller than formerly, and sometimes vacuolar changes could be observed. A pattern of transmigration into normal acinar cells was shown; thus, the tumor suggested including an exocrine-derived component. On immunohistochemical examination (all antibodies for immunohistochemical examination were listed on Table 1), neuroendocrine tumor markers, such as chromogranin A, synaptophysin, and CD56, were positive (Figure 4c/d/e). Some of the tumor cells, particularly in the acinus-like structure, were also positive for α1-antichymotrypsin and α1-antitrypsin (Figure 4f/g). Neurofibromin protein expression was negative in the tumor cells (Figure 4h), in contrast being strongly positive in islets in the non-neoplastic pancreas (Figure 4i). According to the WHO classification (2010), the tumor was diagnosed as “neuroendocrine tumor, NET G2,” because the Mib1 index (%) is 7% and proliferation rate is 4 mitoses per 10HPF [18],and the tumor was composed acinar and neuroendocrine cells. The acinar component was about 15% and neuroendocrine component about 55%; cells with both characterscomprised about 30%. According to the TNM classification [19], the tumor was classified as pT3N1M0, G2, R0, stage IIB. The tumors of the jejunum and stomach were compatible with a diagnosis of gastrointestinal stromal tumor (GIST) presenting with positive expression for c-Kit and CD34 on immunohistochemistry. The patient received adjuvant chemotherapy with TS-1 for 1 year. At follow-up 2 years after surgery, the patient remained well without any recurrent pancreatic disease.

**Table 1:** List of antibodies for immunohistochemical examination

**Antibody**	**Source**
Chromogranin A	Rabbit polyclonal antibody
Synaptophysin	Mouse monoclonal antibody
CD56	Mouse monoclonal antibody
α1-Antichymotrypsin	Rabbit polyclonal antibody
α1-Antitrypsin	Rabbit polyclonal antibody
Neurofibromin	Mouse monoclonal antibody
Ki-67	Mouse monoclonal antibody
c-kit	Rabbit polyclonal antibody
CD34	Mouse monoclonal antibody

## Conclusions

Only 1–2% of cases of PNETs occur as part of an inherited disorder, such as MEN-1, VHL, tuberous sclerosis complex (TSC), and NF-1 [10,11]. The frequency of PNETs with hereditary disease is high in the order of MEN1, VHL, NF-1 and TSC [10]. In patients with NF-1, neuroendocrine tumors sometimes develop: the most common tumor is somatostatinoma arising in the peri-ampullary and duodenal region [6,8,9]. PNET with NF-1 is rare and, to our knowledge, only six cases of PNETs with NF-1 have previously been reported (Table 2) [12-17].

**Table 2:** Pancreatic neuroendocrine tumors in patients with neurofibromatosis-1

**Authors [ref.]**	**Year**	**Sex**	**Age (years)**	**Site of tumor in pancreas**	**Histology**	Malignant*
Coskey and Tranquanda [12]	1964	Female	66	Body and tail	Insulinoma	Yes
Saurenmann et al. [13]	1987	Male	62	Head	Somatostatinoma	No
Fung and Lam [14]	1995	Male	45	Body and tail	Insulinoma	No
Thannberger et al. [15]	2001	Male	28	Head	Somatostatinoma	Yes
Fujisawa et al. [16]	2002	Female	66	Head	Neuroendocrine carcinoma	Yes
Perren et al. [17]	2006	ND	ND	Body	Insulinoma	Yes
Nishi et al. (present case)		Male	62	Head	Neuroendocrine tumor, NET G2	Yes

*These cases were reported before 2010, so it is difficult to apply the new WHO classification (2010). Although our case is NET G2, NET G2 was well-differentiated adenocarcinoma in the previous WHO classification (2000). We listed our tumor as malignant according to the previous diagnosis to check with other cases.

Neurofibromin, the product of the *NF-1* gene, acts as a tumor suppressor because it inhibits the activity of ras guanosine triphosphatase-activating protein, which regulates cell proliferation and differentiation [2,3]. Mutation of the *NF-1* gene and dysfunction of neurofibromin lead to uncontrolled cell proliferation and development of some tumors, including neurofibroma, glomus tumor, carcinoid tumor, and gastrointestinal tumor. The rarity of PNET with NF-1 raises some doubt about whether it occurs coincidentally or because of *NF-1* gene mutation. Perren et al. [17] reported that neurofibromin expression was negative in an insulinoma of the pancreas in a patient with NF-1, but that neurofibromin expression was strongly positive in a sporadic insulinoma on immunohistochemistry; in addition, neurofibromin expression was reduced at the mRNA level in the insulinoma of the pancreas in a patient with NF-1. Thus, they concluded that their case was a result of an *NF-1* gene mutation. Furthermore, Speel et al. [20] analyzed genetic differences in PNETs and found that no sporadic tumors had a loss of heterozygosity in 17q, which encodes the *NF-1* gene. These results indicate that PNET in patients with NF-1 might be induced by the *NF-1* gene mutation. In our case, the PNET was negative for neurofibromin, suggesting a decrease in neurofibromin expression associated with *NF-1* gene mutation. In addition, in our case, hyperplasia of islet cells occurred frequently in the non-neoplastic pancreas, which is a very unusual finding in normal NETs. This suggested that the islet cells in our patient had a tendency to develop hyperplasia frequently, and this may have developed into an endocrine carcinomatumor.

GISTs in the jejunum and stomach were found incidentally during surgery in our patient. A high incidence of GIST arising in patients with NF-1 has been reported previously [21]. In addition, GISTs in patients with NF-1 often develop in the small intestine, and multiple occurrence is common, while it is rare in sporadic GISTs [21]. Furthermore, coincidental development of GIST and NET has been recognized, particularly in patients with NF-1 [22]. Recently, Yamamoto et al. [23] reported that *KIT* and platelet-derived growth factor receptor-alpha (*PDGFRA*) mutations are very rare events in NF-1 GIST and that activation of the RAS-MAPK pathway associated with inactivation of the *NF-1* gene may play an important role in the development of GIST in NF-1 patients.

NF-1 is also associated with various benign and malignant neoplasms, including tumors of the nervous system and gastrointestinal tract. The main cause of death in patients with NF-1 is malignant nerve sheath tumor [24]. Our patient had a PNET (G2) and multiple GISTs, as well as subcutaneous neurofibromas; pancreatic endocrine carcinoma was considered to be the most life-threatening tumor. Of the six reported cases of PNET with NF-1, four were malignant. These cases were reported before 2010, so it is difficult to apply the new WHO classification(2010),although the case reported as carcinoma seems to have malignant features, and is considered NET G2 or NEC. On the other hand, Relles et al. [9] demonstrated that only 1% of peri-ampullary and duodenal NETs in patients with NF-1 were malignant. Therefore, PNET with NF-1 seems to include a great potential for malignancy, and there might be some differences in tumorigenesis between peri-ampullary NETs and PNETs in NF-1 patients. Although our patient remained well without any recurrent pancreatic disease at follow-up 2 years after surgery, careful observation is required.

In summary, PNET arising in patients with NF-1 is a rare occurrence and has the potential to be highly malignant. Although mutation of the *NF-1* gene and dysfunction of neurofibromin may affect on the development of PNET in patients with NF-1, further investigation is required to clarify this association.

## Consent

Written informed consent was obtained from the patient for publication of this case report and any accompanying images. A copy of the written consent is available for review by the Editor in Chief of this journal.

## Abbreviations

EUS: Endoscopic ultrasonography; GIST: Gastrointestinal stromal tumor; MEN-1: Multiple endocrine neoplasia type 1; NF-1: Neurofibromatosis; PNET: Pancreatic neuroendocrine tumor; TSC: Tuberous sclerosis complex; VHL: von Hippel-Lindau.

## Competing interests

The authors declare that they have no competing interests.

## Authors’ contributions

TN carried out the surgical procedure, designed the report, analyzed all the reports, and drafted the manuscript. YK, HY, and SY carried out the surgical procedure and participated in designing the manuscript. HI performed all examinations and made the diagnosis. NI performed the histological analysis of the surgical specimens and provided histological sections as figures for the manuscript. RM performed the histological analysis of the surgical specimens and analyzed the expression of neurofibromin in the immunohistochemistry. YT participated in designing the report and revised the manuscript for submission. All authors have read and approved the final manuscript.
